# Development and Fecundity of Oriental Fruit Moth (Lepidoptera: Tortricidae) Reared on Various Concentrations of Amygdalin

**DOI:** 10.3390/insects13110974

**Published:** 2022-10-24

**Authors:** Yi Wang, Jie Li, Xiaohan Chai, Xuefeng Hu, Xianwei Li, Weina Kong, Ruiyan Ma

**Affiliations:** 1 Shanxi Key Laboratory of Integrated Pest Management in Agriculture, College of Plant Protection, Shanxi Agricultural University, Jinzhong 030801, China; 2 College of Horticulture, Shanxi Agricultural University, Jinzhong 030801, China

**Keywords:** *Grapholita molesta*, amygdalin, life table, concentration-dependent effect, transgenerational comparison

## Abstract

**Simple Summary:**

The Oriental fruit moth (OFM) attacks the fruits and shoots of Rosaceae that contain variable concentrations of amygdalin over the seasonal time. Amygdalin can affect developmental time and fecundity but not feeding or survival of OFM. Diets with amygdalin at low and moderate concentrations promoted faster development and higher fecundity of OFM. OFM reared on 6 mg of amygdalin per g of diet for one or ten generations performed well in terms of r_m_. For OFM, the relationship between amygdalin concentration and host suitability was a biphasic dose-response.

**Abstract:**

*Grapholita molesta* (Busck) (Lepidoptera: Tortricidae), Oriental fruit moth (OFM), attacks fruits and shoots of the economically important trees in Rosaceae. Amygdalin is a cyanogenic glucoside of rosaceous plants that may be related to the seasonal patterns of infestation in many pests. The amygdalin concentration of fruits and shoots of peach, pear, and apple varies over the growing season. However, the relationship between the amygdalin concentration and *G. molesta* performance has not been reported. Here, we measured the performance (feeding, growth, development, and fecundity) of *G. molesta* larvae (as subsequent adults) reared on artificial diets with six amygdalin concentrations (0, 3, 6, 12, 24, and 48 mg/g), and we then calculated the population parameters. We found that these different concentrations of amygdalin affected the developmental time and fecundity, except for the proportion of larvae feeding on the diet and the survival rates of larvae and pupae. When compared with the control diet without amygdalin, diets with 3 or 6 mg/g (low and moderate concentrations) of amygdalin shortened developmental times and increased the number of eggs laid by females; however, a diet with 12 mg/g (moderate concentration) of amygdalin only increased the number of eggs laid by females and did not affect the larval and pupal developmental rate. A diet with 48 mg/g (high concentration) of amygdalin prolonged developmental times and reduced the number of eggs laid by females when compared with the control diet without amygdalin. Furthermore, the intrinsic rate of increase (r_m_) for insects reared on diets with 3 or 6 mg/g (low and moderate concentrations) of amygdalin versus the control diet without amygdalin showed a slightly improved population growth. However, this increase in the r_m_ value did not persist over ten successive generations of rearing on the same diet. We concluded that the diet with 6 mg of amygdalin per g of diet can enhance the performance and population growth of *G. molesta*, but the effects of amygdalin are concentration-dependent.

## 1. Introduction

The Oriental fruit moth, *Grapholita molesta* (Busck) (Lepidoptera: Tortricidae), is a species that bores in and damages economically important fruits and shoots of Rosaceae trees, including apples (*Malus domestica*), peaches (*Prunus persica*), nectarines (*Prunus persica* var. *nectarina*), pears (*Pyrus communis*), apricots (*Armeniaca vulgaris*), quince (*Cydonia oblonga*), almonds (*Amygdalus communis*), plums (*Prunus salicina*), and cherries (*Cerasus pseudocerasus*) [1,2,3]. *G. molesta* has multiple generations per year, which overlap [4,5], and the extent of its damage on shoots and fruits varies in different host plants and moth generations. There are many secondary metabolites that function as defense compounds, such as phenols, flavonoids, and tannins, in the host plants of *G. molesta*, which are toxic, repellent, or anti-nutritional to herbivorous insects [6,7]. These secondary metabolites are also found in other plants and can affect the feeding, growth, development, and reproduction of many moth pests [8,9,10,11,12,13]. For *G. molesta*, previous studies have shown that flavonoids only inhibited fruit-boring of fifth-generation larvae, polyphenols were not related to the fruit-boring preferences of larvae [14], and phenols, tannins, and flavones were not related to the shoot-boring preferences of larvae [15].

The host plants of specialized insects are usually related species or families of plants that contain similar secondary metabolites [16]. In nature, the bitter cyanogenic glucosides (CNGs) serve as good chemotaxonomic markers for plant relatedness [17]. Amygdalin is a kind of CNG found in the rosaceous host plants of *G. molesta* [18,19]. Previous studies have reported that CNGs act as feeding and oviposition deterrents in insects [20]. CNGs are among the most widespread defense chemicals of plants [21,22,23,24]. However, among different insects, responses to CNGs in plants varies from neutrality to avoidance. By adapting to the secondary metabolites of specific plants, insects can successfully form host relationships that allow them to establish enduring populations on the target plants [25]. CNGs are considered as an effective defense against generalist herbivores; however, insects that are specialist feeders on plants with CNGs are generally believed to be adapted to this group of plant defenses [26], and they may even use the plant defenses to promote their own development and reproduction [27,28,29]. Especially among Lepidoptera, many species can thrive on plants defended by CNGs. Specialists may even sequester CNGs from their host plants in their larvae [23,30] or engage in de novo synthesis of CNGs. CNGs in insects can be used as defense compounds against natural enemies or may play a role in mating based on male transfer of CNGs to females [31,32]. For example, some moths in Zygaenidae, Acraeinae, Heliconiinae, Nymphalinae, and Polyommatinae prefer to feed on cyanogenic plants and rely on the available cyanogenic host plants as a major food source. Previous studies have shown that concentrations of amygdalin from different parts of fruits may be as high as 17.49 mg/g in seeds or as low as 0.03 mg/g in fruit pulp, and may vary with season [33,34]. However, the effects of different concentrations of amygdalin on the feeding performance and population growth of *G. molesta* have not been reported.

Therefore, the effects of different amygdalin concentrations on feeding, growth, development, reproduction, and population increase of *G. molesta* were examined in this study. Five amygdalin concentrations presented in an artificial diet were examined as treatments compared to the same diet without amygdalin as the control. The work was carried out in three phases. First, we determined the proportion of larvae feeding on the different artificial diets (i.e., various concentrations of amygdalin). Second, we examined the fate of larvae feed for one generation on each of the six artificial diets amended with different concentrations of amygdalin. Third, we assessed the impact of an artificial diet amended with 6 mg of amygdalin per g of diet over the course of ten successive generations. To determine the effect of different diets on the performance of *G. molesta*, we measured the developmental times, pupal weights, and survival rates of the immature stages, and adult fecundity. We then calculated population parameters for moths reared on each diet. These experiments explore the impacts of secondary metabolites in host plants on the oligophagous moth *G. molesta*.

## 2. Materials and Methods

### 2.1. Insect Rearing

The original population of *G. molesta* used in our experiments was collected from a peach orchard, *Prunus persica* (L.) Batsch cv. Okubao, in Taigu, Shanxi, China (37°180 N, 112°290 E, 824 m above sea level) in 2010. Infested shoots and fruits were collected, and the emerging adults were maintained as a colony for over 50 generations at Shanxi Agricultural University, China. No host plants were used in the rearing process. Larvae of the colony were reared until pupation on 1.5 cm^3^ of an artificial diet (modified from Du et al., 2010) [35], in glass tubes (inner diameter: 1.5 cm, length: 10 cm), plugged with absorbent cotton balls. Insects were reared over their entire life cycle in growth chambers set at 26 ± 0.8 °C, 70–80% r.h., and a 15:9 h L:D photoperiod.

### 2.2. Artificial Diet Preparation

Different amounts (3.29, 6.59, 13.18, 26.35, and 52.71 g) of amygdalin (analytical reagent, purity: 98.0%, Aikeda Reagent Co., Chengdu, China) were added to the artificial diet including 75 g of corn flour, 75 g of soybean meal, 14 g of agar, 30 g of yeast, 3 g of vitamin C, 1 g of sorbic acid, 0.1 g of cholesterol, 2 g of nipagin, 198 g of tomato sauce, and 700 g of distilled water, which was used to maintain the colony of *G. molesta* and the amended diets were used as treatments. The unamended artificial diet (0 mg of amygdalin per g of diet) was the control, and then the mass fractions of amygdalin in the treatment artificial diets were 3, 6, 12, 24, and 48 mg of amygdalin per g of initial artificial diet. The test diets were grouped as low (0 and 3 mg/g), moderate (6 and 12 mg/g), and high (24 and 48 mg/g) based on amygdalin levels.

### 2.3. Larval Feeding: Multiple Choice Test

The six artificial test diets were cut into 0.5 cm^3^ cubes, and one cube of each artificial diet was randomly selected and placed in a plastic petri dish (inner diameter: 6 cm) near the edge, so that each dish contained one cube of each of the six diets. One 2nd or 3rd instar *G. molesta* larva from the colony, which had been reared on the unamended artificial diet (without amygdalin), was placed in the center of the plastic petri dish and allowed to feed. Six replicates (each with 10 larvae spread over 10 dishes) were tested, for a total of 60 larvae in each of the six treatments in this test (=360 together). The number of larvae feeding on each artificial diet was then checked after 24 h.

### 2.4. Performance Measurement over One or Ten Generations

This experiment was carried out in two parts: (1) six artificial diets amended with amygdalin at different levels were used to feed larvae over one generation and (2) two artificial diets amended with 0 or 6 mg of amygdalin per g of diet were used to feed larvae over ten successive generations. In both parts, the unamended artificial diet (0 mg of amygdalin per g of diet) was used as the control.

To obtain neonate larvae, eggs of *G. molesta* of a similar age were collected following the procedure described by Kong et al. (2020) [3]. When embryos in eggs reached the black head stage (about ready to hatch), the waxed paper bearing the eggs was cut into pieces. Waxed paper pieces, each bearing 20 eggs, were transferred individually into glass tubes (inner diameter: 1.5 cm, length: 10 cm) and placed at the bottom of the glass tube. For each treatment or control, 200 eggs (20 eggs in each of ten glass tubes) were tested. Cubes (1.5 cm^3^) of each of the treatment or control diets were selected randomly and placed in glass tubes (one cube per glass tube). The cubes were placed 2/3 of the way along the length of the glass tube and did not touch the waxed paper bearing the eggs.

***Immature stages*.** Although the eggs did not feed the artificial diets, the eggs were examined daily to record the egg hatch and survival rate. Their values were not affected by different amounts of amygdalin (Supplementary Figures S1–S4), thus indicating that 194–200 newly hatched larvae were observed in each of the six treatments. They were fed by artificial diets with six amygdalin concentrations. Larval status was checked daily until all larvae had died or developed to 5th instar [2]. Mature larvae were transferred into separate plastic tubes (inner diameter: 1 cm, length: 10 cm) and held for pupation. Plastic tubes were checked daily to record pupation and adult emergence, and to calculate larval and pupal survival and developmental times. Pupal weight was recorded on day 2 after pupation.

***Mature stage*.** All newly emerged females and males from the same artificial diet were held together in one glass cylinder (high: 28 cm, inner diameter at the two ends: 9 and 17 cm) for mating, which typically occurred at 5:00–10:00 p.m. [36]. Adults were fed 15 mL of a 5% honey solution. The numbers of eggs laid on the surface of the glass cylinders or on the waxed papers placed on the bottom of the cylinders was recorded daily. Each new egg was marked daily with a small circle placed on the outside of the glass cylinders or on the waxed papers. Meanwhile, the numbers and sex of all dead adults were recorded daily. The total numbers of laid and hatched eggs were counted over each female’s whole lifetime. All the containers were maintained in a climate chamber under the same conditions as the colony.

For part one of this experiment, the above observations were made for all 6 diets for one moth generation. For part two, these observations were made rearing on the test diets (at two amygdalin levels) for ten generations.

### 2.5. Statistical Analysis

The proportion of larvae feeding on each of the different artificial diets, the developmental times and survival rates of larvae, pupae, and the entire immature stage, as well as the pupal weights were square-root transformed to stabilize their variances before analysis [37]. One-way ANOVA was used to investigate the data on life history parameters of moths exposed to amygdalin for one or ten successive generation. For developmental time and pupal weight, one larva/pupa was recorded as one replicate (larva: *n* = 44–122 replicates, pupa: *n* = 31–116 replicates). For survival rate, larvae/pupae following emergence from twenty eggs were recorded as one replicate (*n* = 10 replicates). A Shapiro–Wilk test was used to test the normality of the residuals. Means with significant differences were separated using the Tukey’s HSD or the Games–Howell test, depending on whether the variances were equal (Shapiro–Wilk test, *p* > 0.05) or unequal (Shapiro–Wilk test, *p* < 0.05). All data were analyzed in the statistical program SPSS (IBM SPSS v. 19.0, Armonk, NY, USA).

The ratio of the total number of eggs laid to the number of females in the rearing group was used to calculate the number of eggs laid per female.

Life table statistics on (1) the populations feeding on six amygdalin artificial diets for one generation, and (2) the populations feeding on artificial diets amended with 0 and 6 mg of amygdalin per g of diet after ten successive generations were calculated, including the intrinsic rate of increase (r_m_), the finite rate of increase (λ), the net reproductive rate (R_0_), and the mean generation time (T) using POP TOOLS 3.2.5 [38].

## 3. Results

### 3.1. Larval Feeding on Diets and Developmental Times of Immature Stages

**Figure 1:**
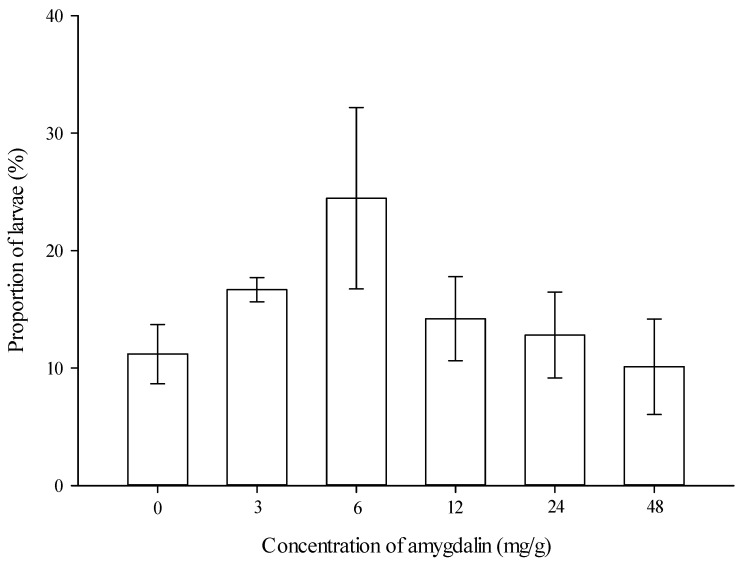
Proportion of *Grapholita molesta* larvae feeding on the artificial diets (mean ± SE) amended with different levels of amygdalin.

***Proportion of larvae feeding on diets.*** The proportion of larvae feeding on the artificial diets was not significantly affected by amygdalin concentrations (*F* = 1.339, df = 5,35, *p* = 0.275) (Figure 1).

**Figure 2:**
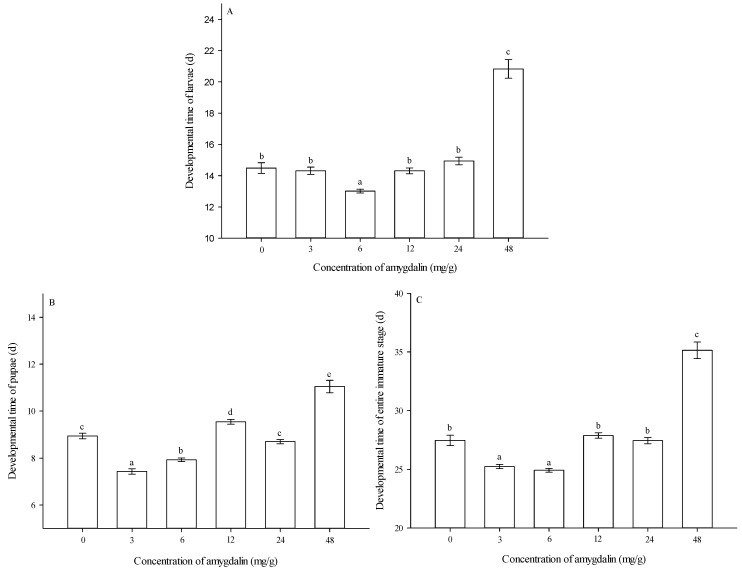
Effects of different amygdalin levels on developmental times (mean ± SE) of *Grapholita molesta* larvae (**A**), pupae (**B**) and entire immature stages (**C**). The different lowercase letters indicate significant differences among different amygdalin levels at *p* < 0.05, based on Tukey’s HSD or Games–Howell test.

***Developmental times of larvae***. The developmental times of larvae were significantly affected by amygdalin concentrations (*F* = 84.298, df = 5624, *p* < 0.05). At 6 mg/g, larval developmental time was shortest among the different concentrations. At 3, 12, and 24 mg/g, larval developmental times were not significantly different from the control (0 mg/g). At 48 mg/g, larval developmental time was significantly longer than that of the control (0 mg/g) (Figure 2A).

**Figure 3:**
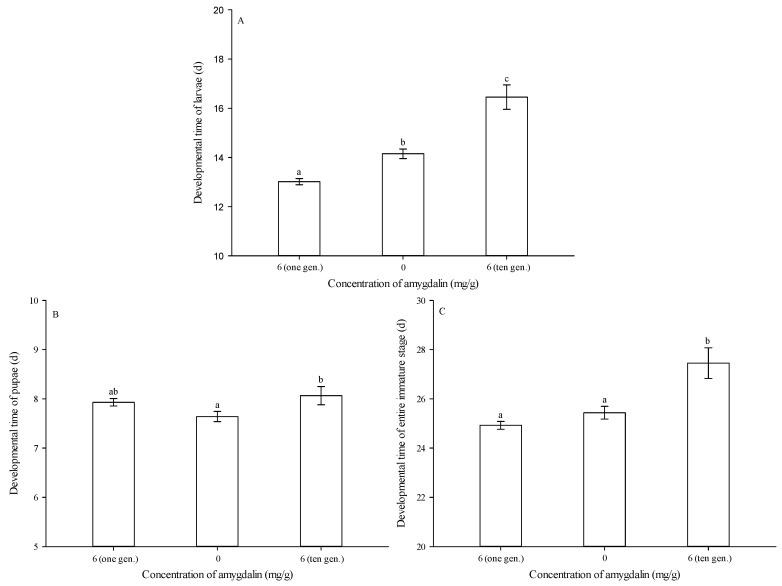
Developmental times (mean ± SE) of *Grapholita molesta* larvae (**A**), pupae (**B**) and entire immature stages (**C**) after ten successive generations of feeding on diets amended with 0 or 6 mg of amygdalin per g of diet. The different lowercase letters indicate significant differences among different amygdalin levels at *p* < 0.05, based on Tukey’s HSD or Games–Howell test.

While, as noted above, the diet amended with 6 mg of amygdalin per g of diet shortened larval developmental time, this effect was reversed over multiple generations of exposure. Specifically, after ten successive generations of feeding on diet amended with 6 mg of amygdalin per g of diet, larval developmental time was significantly longer than that of the control (0 mg/g) and the group reared on 6 mg/g of amygdalin for only one generation (*F* = 47.338, df = 2272, *p* < 0.05) (Figure 3A).

***Developmental times of pupae***. The developmental times of pupae were significantly affected by amygdalin concentrations (*F* = 86.784, df = 5563, *p* < 0.05). At 3 and 6 mg/g, pupal developmental times were significantly shorter than that of the control (0 mg/g). At 24 mg/g, pupal developmental time showed no significant difference from the control (0 mg/g). At 12 and 48 mg/g, pupal developmental times were significantly longer than that of the control (0 mg/g) (Figure 2B).

After ten successive generations of feeding on artificial diet amended with 6 mg of amygdalin per g of diet, pupal developmental time was significantly longer than that of the control (0 mg/g), but it was not significantly different from that of group reared on 6 mg/g of amygdalin for only one generation (*F* = 3.879, df = 2235, *p* = 0.022) (Figure 3B).

***Developmental times of the entire immature stage***. Developmental times of the entire immature stage were significantly affected by amygdalin concentrations (*F* = 108.136, df = 5563, *p* < 0.05). At 3 and 6 mg/g, developmental times of the entire immature stage were significantly shorter than that of the control (0 mg/g). At 12 and 24 mg/g, developmental times of the entire immature stage were not significantly different from the control (0 mg/g). At 48 mg/g, developmental time of the entire immature stage was significantly longer than that of the control (0 mg/g) (Figure 2C).

After ten successive generations of feeding on an artificial diet amended with 6 mg of amygdalin per g of diet, developmental time of the entire immature stage was significantly longer than that of the control (0 mg/g) and the group reared on 6 mg/g of amygdalin for only one generation (*F* = 14.572, df = 2235, *p* < 0.05) (Figure 3C).

### 3.2. Survival Rates of Immature Stages

**Figure 4:**
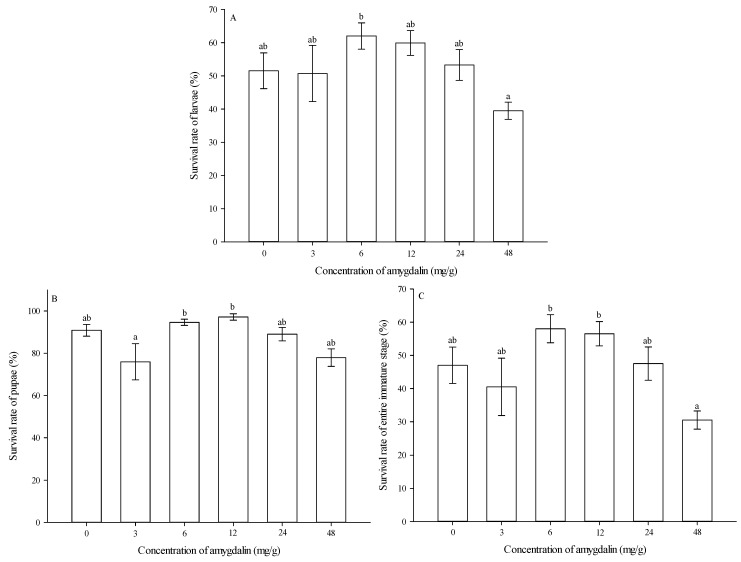
Effects of amygdalin levels on survival rates (mean ± SE) of *Grapholita molesta* larvae (**A**), pupae (**B**) and entire immature stages (**C**). The different lowercase letters indicate significant differences among different amygdalin levels at *p* < 0.05, based on Tukey’s HSD or Games–Howell test. Number of pupae following emergence from larvae fed by different amygdalin concentrations (0, 3, 6, 12, 24, and 48 mg/g) were 102, 101, 122, 116, 106, and 78. Number of adults following emergence from larvae fed by different amygdalin concentrations (0, 3, 6, 12, 24, and 48 mg/g) were 94, 81, 116, 113, 95, and 65.

***Survival rates of larvae***. Survival rates of larvae were significantly affected by amygdalin concentrations (*F* = 2.407, df = 5,59, *p* = 0.048). At 6 mg/g, larval survival rate was significantly higher than that at 48 mg/g. However, the larval survival rates were not significantly different from that of the control (0 mg/g) at any amygdalin level (Figure 4A).

**Figure 5:**
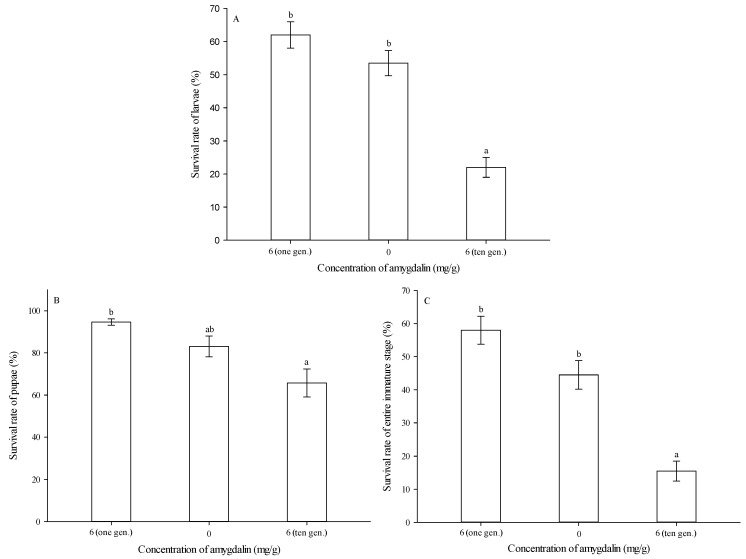
Survival rates (mean ± SE) of *Grapholita molesta* larvae (**A**), pupae (**B**) and entire immature stages (**C**) after ten successive generations of feeding on diets amended with 0 or 6 mg of amygdalin per g of diet. The different lowercase letters indicate significant differences among different amygdalin levels at *p* < 0.05, based on Tukey’s HSD or Games–Howell test. Numbers of pupae following emergence from larvae fed by 6 mg/g of amygdalin for one generation, 0 and 6 mg/g of amygdalin for ten generations were 122, 107, and 44. Numbers of adults following emergence from larvae fed by 6 mg/g of amygdalin for one generation, 0 and 6 mg/g of amygdalin for ten generations were 116, 89, and 31.

After ten successive generations of feeding on an artificial diet amended with 6 mg of amygdalin per g of diet, larval survival rate was significantly lower than that of either the control (0 mg/g) or the group reared on 6 mg/g of amygdalin for only one generation (*F* = 35.329, df = 2,29, *p* < 0.05) (Figure 5A).

***Survival rates of pupae***. Survival rates of pupae were significantly affected by amygdalin concentrations (*F* = 3.568, df = 5,59, *p* = 0.007). At 6 and 12 mg/g, pupal survival rates were significantly higher than that at 3 mg/g. However, the pupal survival rates were not significantly different from that of the control (0 mg/g) at any amygdalin level (Figure 4B).

After ten successive generations of feeding on an artificial diet amended with 6 mg of amygdalin per g of diet, pupal survival rate was significantly lower than that for the group reared on 6 mg/g of amygdalin for only one generation, but it was not significantly different from the control (0 mg/g) (*F* = 9.023, df = 2,29, *p* = 0.001) (Figure 5B).

***Survival rates of the entire immature stage***. Survival rates for the entire immature stage were significantly affected by amygdalin concentrations (*F* = 3.782, df = 5,59, *p* = 0.005). At 6 and 12 mg/g, survival rates of the entire immature stage were significantly higher than that at 48 mg/g. However, the survival rates of the entire immature stage were not significantly different from that of the control (0 mg/g) at any amygdalin level (Figure 4C).

After ten successive generations of feeding on an artificial diet amended with 6 mg of amygdalin per g of diet, the survival rate of the entire immature stage was significantly lower than that of either the control (0 mg/g) or the group reared on 6 mg/g of amygdalin for only one generation (*F* = 33.432, df = 2,29, *p* < 0.05) (Figure 5C).

### 3.3. Pupal Weights, Fecundity of Adult Stage and Population Parameters

***Pupal weights***. Pupal weight was significantly affected by amygdalin concentrations (*F* = 3.969, df = 5624, *p* = 0.001). At 3, 6, 12, and 48 mg/g, the pupal weights were not significantly different from the control (0 mg/g). Oddly, pupal weight was depressed at 24 mg/g, but not at 48 mg/g (Table 1).

After ten successive generations of feeding on an artificial diet amended with 6 mg of amygdalin per g of diet, pupal weight was significantly lower than that of the control (0 mg/g), but it was not significantly different from that of group reared on 6 mg/g of amygdalin for only one generation (*F* = 19.11, df = 2272, *p* < 0.05) (Table 2).

***Total number of eggs laid***. The total numbers of eggs laid per group (which were reared on different diets amended with amygdalin) increased by 14%, 58%, 52%, and 14% at 3, 6, 12, and 24 mg of amygdalin per g of diet compared to the control (0 mg/g). The total numbers of eggs laid decreased by 70% at 48 mg/g compared to the control (0 mg/g) (Table 1).

After ten successive generations of feeding on an artificial diet amended with 6 mg of amygdalin per g of diet, the total number of eggs laid per group had decreased by 55% compared to the control (0 mg/g) and 74% compared to the group reared on 6 mg/g of amygdalin for only one generation, which was strongly affected by survival in the larval and pupal stages (Table 2).

***Number of eggs laid per female***. The numbers of eggs laid per female (removing the influence of immature stage survival) increased by 31%, 17%, 13%, and 19% at 3, 6, 12, and 24 mg of amygdalin per g of diet compared to the control (0 mg/g). The number of eggs laid per female decreased by 64% at 48 mg/g compared to the control (0 mg/g) (Table 1).

After ten successive generations of feeding on an artificial diet amended with 6 mg of amygdalin per g of diet, the number of eggs laid per female increased by 47% compared to the control (0 mg/g) and 32% compared to the group reared on 6 mg/g of amygdalin for only one generation (Table 2).

***Population parameters*.** The maximum intrinsic rate of increase (r_m_) occurred at 3 mg of amygdalin per g of diet. Populations fed on the artificial diet amended with 3 or 6 mg/g of amygdalin showed increases in their r_m_ values of 5.15 and 4.15%, whereas populations fed on the artificial diet amended with 12, 24, or 48 mg/g of amygdalin showed reductions in their r_m_ values of 5.88, 7.15, and 39.75%, respectively, compared to the control (0 mg/g). The finite rate of increase (λ) had a pattern similar to that of r_m_ values. The maximum net reproductive rate (R_0_) occurred at 6 mg of amygdalin per g of diet. Populations fed on artificial diets amended with 6 or 12 mg/g of amygdalin showed increases in their R_0_ values of 9.62 and 6.22%, whereas populations fed on artificial diets amended with 3, 24, or 48 mg/g of amygdalin showed reductions in their R_0_ values of 18.84, 21.20, and 57.86%, respectively, compared to the control (0 mg/g). The minimum mean generation time (T) occurred at 3 mg of amygdalin per g of diet. Populations fed on an artificial diet amended with 3 and 6 mg/g of amygdalin showed reductions in their T values (i.e., shortened generation times) of 10.07 and 2.74%, whereas populations fed on artificial diets amended with 12, 24, and 48 mg/g of amygdalin showed increases in their T values (i.e., longer generation times) of 8.33, 0.35, and 27.78%, respectively, compared to the control (0 mg/g) (Table 3).

After ten successive generations of feeding on an artificial diet amended with 6 mg of amygdalin per g of diet, the intrinsic rate of increase (r_m_), the finite rate of increase (λ), the net reproductive rate (R_0_), and the mean generation time (T) were all relatively lower compared with that of moths fed on the control (0 mg/g). Furthermore, the values of r_m_ and λ for moths reared on this diet for ten generations were relatively higher compared with that of moths reared on the same diet for only one generation, whereas the values of R_0_ and T for the ten-generation group were relatively lower when compared with the one-generation group (Table 4).

## 4. Discussion

Larvae of *G. molesta* normally feed on the shoots of peach in the early season and then the fruits of peach, apple, and pear later in the season [39,40,41]. Previously, we reported that amygdalin levels in peach shoots are higher (3.14 mg/g) in April than in other host plants (<1 mg/g, shoots of pear and apple in April, and fruits of peach, pear, and apple in May to August) [34]. In the present study, we found that the artificial diets amended with amygdalin at different concentrations affected the developmental time and fecundity but not the proportion of larvae feeding on the diets or larval and pupal survival rate, findings that were similar to those for *G. molesta* and *Cydia pomonella* (L.) (Lepidoptera: Tortricidae) feeding on artificial diets amended with juglone [42,43].

When compared with the unamended control, the developmental times of larvae, pupae, or entire immature stage were shorter on artificial diets amended with 3 or 6 mg/g (low and moderate concentrations) of amygdalin, whereas the developmental time increased on artificial diets amended with 48 mg/g (high concentration) of amygdalin. This is supported by previous reports that the development of some Lepidoptera was affected by some doses of secondary metabolites [e.g., *Lymantria dispar* (L.), *G. molesta*, *Hyphantria cunea* (Drury)] [11,12,42,44]. Typically, cyanogenic glycosides (e.g., linamarin, lotaustralin) and glucosinolate promoted immature stage growth of *Zygaena filipendulae* (L.) [45] and of diamondback moth, *Plutella xylostella* (L.) [46], respectively. Insects experience shortened developmental times on high quality food but compensate on low quality hosts by increasing the developmental time [47,48]. Our results here indicated that diets amended with 3 or 6 mg/g (low and moderate levels) of amygdalin were favorable for *G. molesta* development.

When compared with the unamended control, the number of eggs laid per female after larvae were fed 3 mg/g (low concentration) of amygdalin and the total number of eggs (incorporating survival of immatures in test group) laid after larvae were fed 6 or 12 mg/g (moderate concentrations) of amygdalin increased by more than 30% and 50%, respectively. Enhancement of fecundity by secondary metabolites in Lepidoptera was reported for *P. xylostella* [46]. However, if larvae were fed on diets with high levels of amygdalin (48 mg/g), the number of eggs laid per female or the total number of eggs (including effects on immature stage survival) laid decreased by more than 60% and 70%, respectively. The inhibition on fecundity by plant secondary metabolites was reported for *Spodoptera littoralis* (Boisduval) [49]. The effect of amygdalin on fecundity of *G. molesta* may be an example of hormesis, in which low-doses promote a function but high-doses inhibit it [50,51,52,53]. Thus, in our case, low and moderate doses of amygdalin (3, 6, and 12 mg/g) promote fecundity, but a high does (48 mg/g) inhibits it in *G. molesta* moths.

Life table parameters, especially the intrinsic rate of increase (r_m_), are often used to integrate all effects of a factor on species population growth [54]. For larvae fed artificial diets with different concentrations of amygdalin, all observed values of r_m_ suggested expanding populations [3]. When larvae were fed an artificial diet with a high level of amygdalin (48 mg/g), the r_m_ value obtained indicated a decrease of 40% in the population growth relative to the control (0 mg/g). However, the variation in r_m_ of all other concentrations versus 0 mg/g of amygdalin was very low (4–7%). Similarly, high levels of glucosinolate are associated with increased levels of r_m_ for the diamondback moth, *P. xylostella* [46]. Furthermore, although the dose-dependent effect of some secondary metabolite on insect life history parameters has been observed, herbivore counteradaptation to such compounds may not occur over the whole concentration range found in potential host plants [43,55]. In our study system, the range of amygdalin tested was similar to but then exceeded levels that occur in suitable host plants of *G. molesta*.

In our study, when *G. molesta* larvae were fed on an artificial diet amended with 6 mg of amygdalin per g of diet for ten generations, the resulting r_m_ value (0.106) indicated that this diet was suitable for population growth of *G. molesta*, mainly because of the increasing number of eggs laid per female. We did find differences in growth, development, and fecundity between feeding larvae the diet for one generation versus ten successive generations, except for r_m_, which remained the same.

Our results showed that amygdalin promoted development and fecundity of *G. molesta* at rates of 3, 6, and 12 mg/g (low to moderate concentrations). Importantly, the optimum concentration of amygdalin was 6 mg/g (moderate concentration) for growth, development, and fecundity. The amygdalin level we found to be optimal (6 mg/g) in our study was close to that found in peach kernels (6.81 mg/g) [33]. Similarly, the different glucosinolates tested incorporated in artificial diets also act in a dose-dependent manner on herbivores, with stimulating effects at low levels and acting as a deterrent at high concentrations [56,57,58,59], and resulting in optimal performance at intermediate levels [55,60]. Our findings are in line with previous studies reporting biphasic dose-responses, with a secondary metabolite acting as a plant allomone at high concentration, but a kairomone for pest herbivores at low concentration [16,61]. This pattern may be related to the enzyme activity in insects. Higher trehalase activity has been reported as the reason why the development of *Spodoptera frugiperda* (Smith) was not impaired by diets amended with 1% amygdalin [62]. Similarly, the level of esterase activity in *Papilio glaucus* (L.) allows its larvae to feed on plants with high levels of phenolic glycosides [63]. Future work should focus on exploring the reasons for adaptation to amygdalin by *G. molesta*.

## Figures and Tables

**Table 1:** Effects of amygdalin concentration on pupal weight (mean ± SE) and *Grapholita molesta* fecundity.

Amygdalin (mg Compound/g Diet)	Pupal Weight (mg)	Total Eggs (Number of Emerged Females in Group)	Number of Eggs Laid/Female
0	8.70 ± 0.16 b	2274 (46)	49.43
3	8.10 ± 0.30 ab	2599 (40)	64.98
6	8.80 ± 0.18 b	3598 (62)	58.03
12	8.60 ± 0.19 ab	3453 (62)	55.69
24	7.90 ± 0.20 a	2595 (44)	58.98
48	9.00 ± 0.27 b	676 (38)	17.79

Notes: The different lowercase letters indicate significant differences among different amygdalin levels at *p* < 0.05, based on Tukey’s HSD or Games–Howell test.

**Table 2:** Pupal weight (mean ± SE) and fecundity of *Grapholita molesta* after one or ten successive generations of rearing on diets with 0 or 6 mg of amygdalin per g of diet.

Amygdalin (mg Compound/g Diet)	Pupal Weight (mg)	Total Eggs Laid (Number of Emerged Females in Group)	Number of Eggs Laid/Female
6 mg/g (1 gen.)	8.80 ± 0.18 a	3598 (62)	58.03
0 mg/g	10.20 ± 0.23 b	2043 (39)	52.38
6 mg/g (10 gen.)	7.90 ± 0.41 a	922 (12)	76.83

Notes: The different lowercase letters indicate significant differences among different amygdalin levels at *p* < 0.05, based on Tukey’s HSD or Games–Howell test.

**Table 3:** Population parameters of *Grapholita molesta* reared on diets with different amygdalin levels.

Amygdalin (mg Compound/g Diet)	r_m_	λ	R_0_	T
0	0.101	1.107	35.431	34.147
3	0.106	1.112	28.756	30.708
6	0.105	1.111	38.838	33.212
12	0.095	1.100	37.633	36.990
24	0.094	1.099	27.920	34.267
48	0.061	1.063	14.930	43.632

Notes: r_m_: Intrinsic rate of increase, λ: Finite rate of increase, R_0_: Net reproductive rate, T: Mean generation time.

**Table 4:** Population parameters of *Grapholita molesta* after ten successive generations of rearing on diet with 0 or 6 mg of amygdalin per g of diet.

Amygdalin (mg Compound/g Diet)	r_m_	λ	R_0_	T
6 mg/g (1 gen.)	0.105	1.111	38.838	33.212
0 mg/g	0.107	1.113	28.635	30.596
6 mg/g (10 gen.)	0.106	1.112	23.978	29.178

Notes: r_m_: Intrinsic rate of increase, λ: Finite rate of increase, R_0_: Net reproductive rate, T: Mean generation time.

## Data Availability

The data presented in this study are available on request from the corresponding author.

## Supplementary Materials

The following supporting information can be downloaded at: https://www.mdpi.com/article/10.3390/insects13110974/s1, Figure S1: Developmental time of eggs (mean ± SE) used to determine the effect of amygdalin concentrations on *Grapholita molesta* for one generation; Figure S2: Developmental time of eggs (mean ± SE) used to determine the effect of ten successive generations of feeding on artificial diet amended with 6 mg/g of amygdalin on *Grapholita molesta*; Figure S3: Survival rate of eggs (mean ± SE) used to determine the effect of amygdalin concentrations on *Grapholita molesta* for one generation; Figure S4: Survival rate of eggs (mean ± SE) used to determine the effect of ten successive generations of feeding on artificial diet amended with 6 mg/g of amygdalin on *Grapholita molesta*.
