# Supplementary material for: Development and Fecundity of Oriental Fruit Moth (Lepidoptera: Tortricidae) Reared on Various Concentrations of Amygdalin
Source: Insects. 2022 Oct 24;13(11):974. doi: 10.3390/insects13110974 (PMC9694010; doi:10.3390/insects13110974)
Supplement: Supplementary file 1 [file insects-13-00974-s001.zip › insects-1980952-supplementary.pdf]

Supporting Information

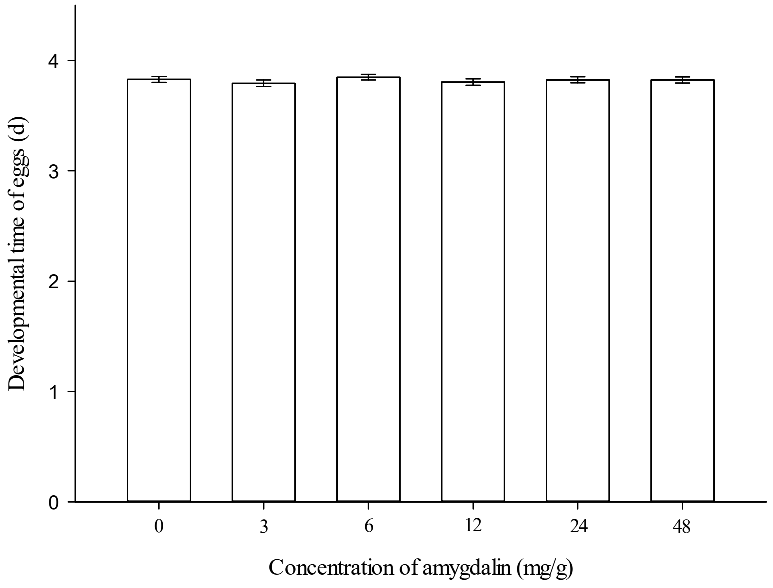

Figure S1. Developmental time of eggs (mean ± SE) used to determine the effect of amygdalin concentrations on *Grapholita molesta* for one generation.

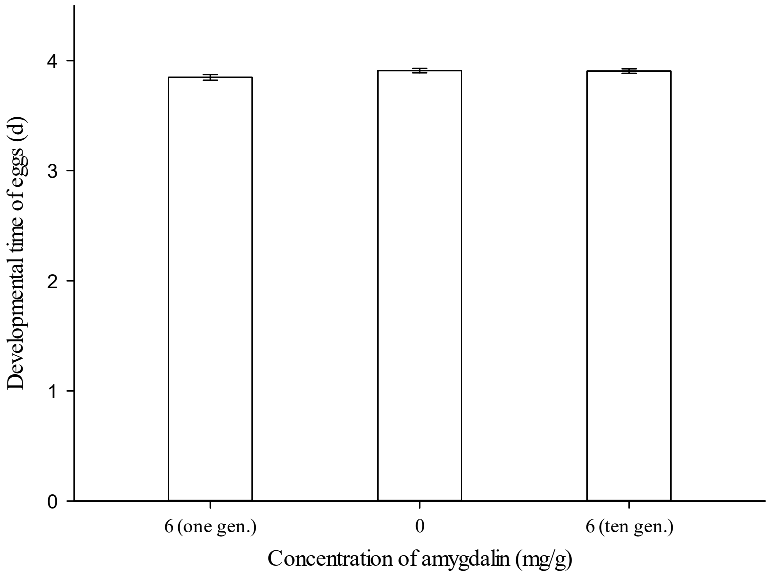

Figure S2. Developmental time of eggs (mean ± SE) used to determine the effect of ten successive generations of feeding on artificial diet amended with 6 mg/g of amygdalin on *Grapholita molesta*.

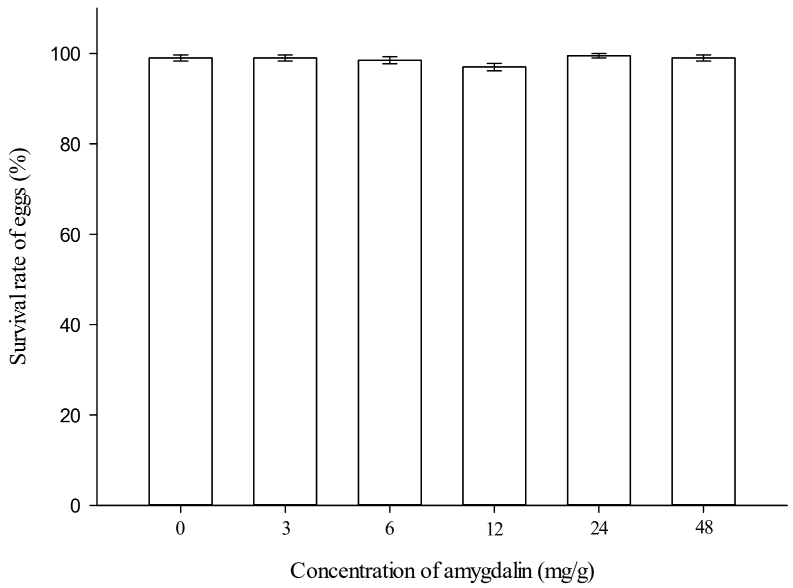

Figure S3. Survival rate of eggs (mean ± SE) used to determine the effect of amygdalin concentrations on *Grapholita molesta* for one generation.

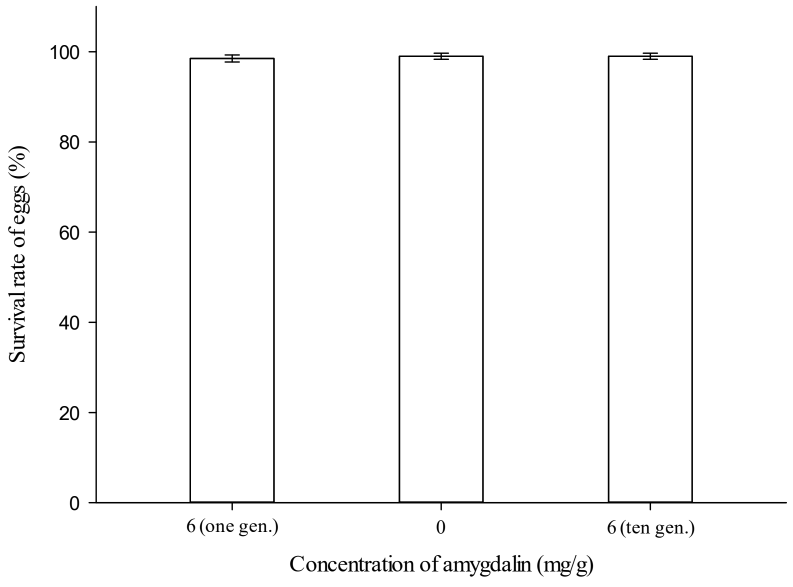

Figure S4. Survival rate of eggs (mean ± SE) used to determine the effect of ten successive generations of feeding on artificial diet amended with 6 mg/g of amygdalin on *Grapholita molesta*.
